# The Risk of Salt Reduction in Dry-Cured Sausage Assessed by the Influence on Water Activity and the Survival of *Salmonella*

**DOI:** 10.3390/foods11030444

**Published:** 2022-02-02

**Authors:** Luis Patarata, Liliana Fernandes, José António Silva, Maria João Fraqueza

**Affiliations:** 1 CECAV, Centro de Ciência Animal e Veterinária, Universidade de Trás-os-Montes e Alto Douro, 5001-801 Vila Real, Portugal; li.li.li.car.5566@gmail.com (L.F.); jasilva@utad.pt (J.A.S.); 2 CIISA, Centro de Investigação Interdisciplinar em Sanidade Animal, Faculdade de Medicina Veterinária, Universidade de Lisboa, Avenida da Universidade Técnica, 1300-477 Lisbon, Portugal; mjoaofraqueza@fmv.ulisboa.pt

**Keywords:** water activity, aw, dry-cured sausages, *chouriço*, salt, phosphates, wine, *Salmonella*

## Abstract

Water activity (aw) is the main hurdle for microbial control in dry-cured sausages. The aw can be influenced by drying or adding electrolytes or humectants. Dry-cured meat products are partially dried, which, together with added salt, results in safe aw values. Currently, there is a trend to reduce salt in meat products, which can compromise the preservation process. The present work aims to evaluate the influences of added salt levels (1% or 3%) and the use or omission of phosphates and wine on the aw of a dry-cured sausage, and to evaluate the possibility of estimating the aw from the moisture loss and the behavior of *Salmonella* during dry-cured sausage (*chouriço*) processing. There was a strong relationship between moisture and aw, regardless of the salt level and the presence of phosphates or wine. Predicting aw from moisture loss is possible using the Boltzmann sigmoid function. The salt level strongly influences *Salmonella* behavior, mainly through aw reduction. An increase in aw by 0.01 units reduced the odds of achieving a 5-log reduction in *Salmonella* counts to half. Increasing added salt from 1% to 3% increased the odds of achieving a 5-log *Salmonella* reduction 7.5-fold. The current trend to reduce salt in foods must be carefully approached if applied to cured meat products, as it has substantial consequences on aw evolution and *Salmonella* survival.

## 1. Introduction

The water activity (aw) of dry sausages, whether cured or fermented, is the main hurdle for pathogen and spoilage microorganism control. Dry meat products are intermediate moisture foods (IMFs) due to their typical aw between 0.60 and 0.90. Their preservation relies on an aw below 0.91, or slightly higher if the pH is low enough [1,2]. The other hurdles included in these meat products are the low temperature used in the early phases of processing and the presence of a competitive microbiota, chemical additives, antimicrobial compounds of spices, smoke, and other ingredients [3,4]. *Chouriço* is a cured meat product usually made with wine during seasoning; particularly in wine-producing regions, meat and fat are cut into small pieces. It is cold-smoked and dried until it reaches an aw below 0.91 [5].

From a practical point of view, aw measures the amount of water available for microbial metabolism. The pathogens of concern in the food industry cannot grow or produce toxins below an aw of 0.91. Therefore, most dry-cured meat products can be stored without refrigeration [6,7,8]. Among the pathogens considered biological hazards in dry-cured sausages, *Salmonella* represents a serious concern due to its resistance to some preservation hurdles, competition ability, and low infectious doses [9].

The aw of meat products can be manipulated by drying or adding compounds to bind water and reduce its availability [10]. In dry-cured meat products, the product is partially dried, which, together with added salt, drives the product to safe aw values [11,12]. Other ingredients might also contribute to aw depression. It is common to use wine to season dry-cured sausages in wine-producing regions [13]. Wine might influence the aw by different modes. On the one hand, as it is mostly water, it might increase the aw. On the other hand, wine might reduce the aw due to ethanol, organic acids, and other minor components [14,15].

Phosphates are used to increase the water-holding capacity (WHC), which might seem inconsistent in dry sausage manufacturing [16]. It is used to counteract the exudative tendency of pork meat resulting from young animals or pale, soft, and exudative (PSE) conditions [17]. Phosphates increase the WHC, and therefore can increase the aw due to the higher amount of water retained. However, due to the stronger bond to the proteins, it might also reduce the water available. Additionally, the phosphate molecule itself binds water, having a marginal effect on aw reduction [18,19]. The final aw is the result of this equilibrium between the amount of water held inside the protein matrix, the strength of the water–protein bond, and the presence of compounds that can attract the water molecule, reducing its availability [2,20].

Drying the cured sausages might involve adequate equipment with controlled temperature, relative humidity, and air velocity. Some smaller industries and workshops rely on the natural temperature and relative humidity [21]. The drying level is a compromise between safety, yield, and texture. Eventually, the aw is reduced by increasing the salt level or other water-binding compounds [22]. This is the reason why cured meat products usually have a high salt content. Due to the growing health concerns related to excess salt in the diet, there has been a trend to reduce the salt content in cured meat products [23]. This reduction might jeopardize the safety of dry-cured sausages if drying is not prolonged enough or other hurdles are introduced [24]. Moreover, the complex interactions between water, salt, and other compounds that affect aw depression might be disrupted if the salt content is reduced [25].

For a specific formulation, aw is highly related to the loss of moisture by drying, and the loss of moisture is most of the product’s weight loss [26]. Direct aw measurement is the method used by the industry to monitor the effectiveness of drying; however, small industries and workshops in mountainous regions do not have the equipment or expertise to conduct this with reliability. In these cases, the liberation of the product is estimated by weight loss or by palpation. This informality might result in the liberation of products with a still-unsafe aw. Producers might also face economic losses due to the excess drying. The possibility of estimating the aw from the moisture loss, with more precision than the informal decision made in several small industries and workshops, could help these producers to have a better perception of their dry-cured sausage aw.

The present work aims to evaluate the influence of added salt levels (1% or 3%) and the use or omission of phosphates and wine (1) on the aw of a dry-cured sausage, as well as to evaluate the possibility of estimating the aw from the moisture loss, and the behavior of *Salmonella* during dry-cured sausage (*chouriço*) processing.

## 2. Materials and Methods

### 2.1. Dry-Cured Sausage Preparation

The experiment was conducted with a dry-cured sausage, *chouriço*, commonly made with wine-marinated meat. This practice, strongly associated with wine-producing regions in Portugal and other regions, is carried out mainly for sensory reasons. Samples were analyzed at ten sampling times during processing: before stuffing; after smoking; and at 1, 4, 8, 12, 17, 24, 32, and 46 days of drying. We prepared eight possible combinations from the three studied effects (Table 1).

*Chouriço* was prepared with a commercial crossbred pork belly and shoulder (1:1) obtained at a local market. The meat was analyzed for the presence of *Salmonella* before preparing the inoculated sausages. *Salmonella* was not detected in any of the five samples tested. The meat was thawed at 4 °C to prepare the sausages. The base formulation of *chouriço* included dry garlic powder (0.5%), nitrite, and nitrate (125 mg/kg NaNO_2_ and 125 mg/kg KNO_3_), mixed in salt at 5% each. *Chouriço* was prepared according to previous works [13]. Samples were dried at 15 ± 1 °C with an initial relative humidity (RH) of 90% and consecutive reductions to maintain the RH at 5% below the aw. The experiment included three batches. One sausage from each batch was withdrawn at each sampling time and analyzed in duplicate. 

### 2.2. Water Activity, Moisture, and pH

The sausages were homogenized in a domestic grinder (Moulinex, Lisbon, Portugal). The aw was measured in 10 g of the sample in a Hygroscope DT apparatus with a WA40 probe (Rotronic, Bassersdorf, Switzerland). The moisture content was determined by weight loss at 105 °C until constant weight [27]. The pH was measured directly in the minced samples using a pH meter (model MicropH 2002, Crison, Barcelona, Spain).

### 2.3. Behavior of Salmonella

The suspension for inoculation of *Salmonella* in the cured sausage consisted of a mixture of a reference strain (CECT 4155) and three strains from meat products or their production environment (laboratory collection). The inoculum was prepared as previously described [28]. The inoculation level was approximately 7 log cfu/g, transferred in 10 mL of 0.85% NaCl. Independent inoculum preparations were prepared for each of the three replicates. Samples were taken at the defined times (see Section 2.1).

An initial dilution of 1:10 was prepared in 0.85% NaCl. Serial dilutions were inoculated in CHROMagar *Salmonella* (CHROMagar, Paris, France) followed by incubation at 37 °C for 24 to 48 h. The results are expressed as log cfu/g.

### 2.4. Data Analysis

The effects of salt, phosphates, and wine on the aw, moisture content, and pH were evaluated by analysis of variance. The localization of differences between means was made using the Tukey_HDS (*p* < 0.05). The relationship between moisture and aw was established through nonlinear regression. The Boltzmann sigmoid function was found to have the best-fitting parameters. A set of external data from similar products [29,30] was used to evaluate the potential use of the equations to predict the aw from the moisture. The fitting quality was evaluated by the determination coefficients (R^2^) and RMSE (XLStat, Addinsoft, Paris, France).

To evaluate the contribution of each parameter to *Salmonella* reduction, we prepared a logistic regression model to predict the contribution of aw, salt, phosphates, and wine to meet the reduction criteria of 5 log proposed by the USDA FSI [31] and to achieve 2-log reduction units. The reference class was the lower amount, or absence, of categorical variables (salt, phosphates, and wine). The continuous variable aw required modification (multiplied by 100) once the logistic regression algorithm assumed an increase of one unit in the predictor, and the aw range was only one unit. Receiver operating curves (ROCs) were prepared for each situation (2- or 5-log reduction) [32]. Computation of data was performed using XLStat software (Addinsoft, Paris, France).

## 3. Results and Discussion

### 3.1. Effect of Salt Level, Phosphates, and Wine on aw, Moisture and pH

**Figure 1:**
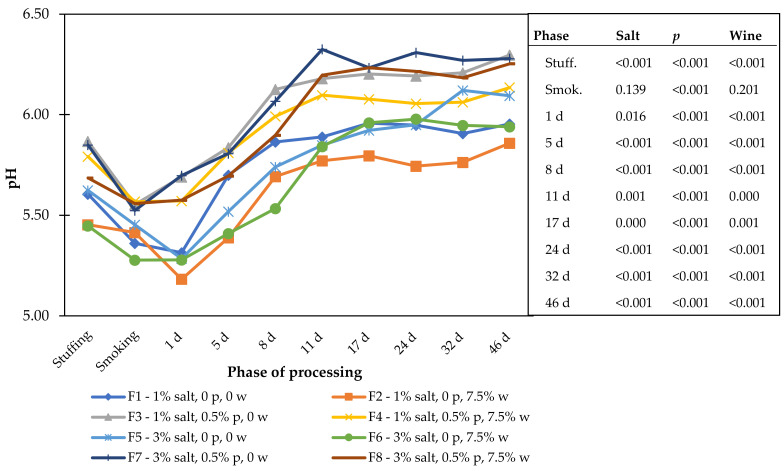
pH in cured sausages with different formulations during processing (p—phosphate; w—wine).

The aw and moisture results are presented in Table 2, and the pH is shown in Figure 1. The first phase of processing analyzed was the stuffing phase. Before that, the meat rested with the seasonings and additives for 24 h at 4 °C. In this phase, the aw mean values were approximately 0.95 and were not influenced (*p* > 0.05) by any of the studied effects. For the duration of the processing, the aw decreased faster in formulations with the higher amount of salt. The use of phosphates had a curious effect. In specific formulations and phases of processing, it contributed to aw reduction, but the opposite effect was observed in other phases. For example, at 17 days of drying, samples containing phosphate had a similar or lower aw than non-phosphate samples. In contrast, at 32 days of drying, samples with phosphates without wine had higher aw values than when the additive was not present. Once in this drying stage, the level of water was already reduced, and the effect of phosphates on aw [19] was more noted than in previous phases.

Almost all formulations achieved safe aw values (≤0.91) on the 17th day of drying, except the sausages made with 1% salt, no phosphate, and 7.5% wine. Drying was prolonged to lower aw values to obtain a broader range of data to be used in the establishment of the relationship between moisture loss and aw, and because these samples were also involved in a microbial challenge test, the extraordinary reduction in aw was necessary to obtain data on the behavior of the pathogen. The effect of wine was not detected in the early drying stages and became significant from the 11th day of drying, with a single difference in sausages with 1% salt and phosphates added. After 17 days of drying, wine addition generally resulted in a lower aw, except in those sausages made with 1% salt and no phosphates. During the last part of drying, wine was revealed to have opposite or neutral effects. It is not easy to extract a pattern from the effect of wine, mainly because wine and phosphates have opposite effects on meat WHC. While phosphates increase the sausage pH [18], wine is acidic and lowers it [33]. Additionally, both can reduce aw by directly binding water molecules; the phosphate itself, and wine, with ethanol and organic acids, may also play a chemical role in water attraction [34,35].

The effect of salt on the moisture content was variable. At the smoking phase, its effect was significant, but the single difference between similar formulations was present in samples with wine and phosphate, which had lower moisture when more salt was added. From the fifth day of drying until the end, samples with more salt generally had higher moisture. The use of phosphate, mainly when no wine was added to the sausage preparation, resulted in higher moisture content. This effect was evident at the beginning of the experiment, where phosphates were responsible for nearly 10% more moisture in the sausages without wine. When the wine was present, the differences were still high, approximately 8% in low-salt sausages, but the differences were not significant in sausages with high salt content. After smoking, the trend of higher moisture in the presence of phosphates was generally maintained.

All the effects under study influenced the pH (Figure 1), with significant differences in almost all processing phases. The use of phosphates promoted a slight increase in pH, and the wine had a slight reduction. The effect of salt, still statistically significant in several processing phases, is not clear enough to establish a pattern. The evolution of pH during processing has a typical pattern of an initial decrease followed by an increase after the beginning of drying [36].

### 3.2. Relationship between aw and Moisture Loss

**Figure 2:**
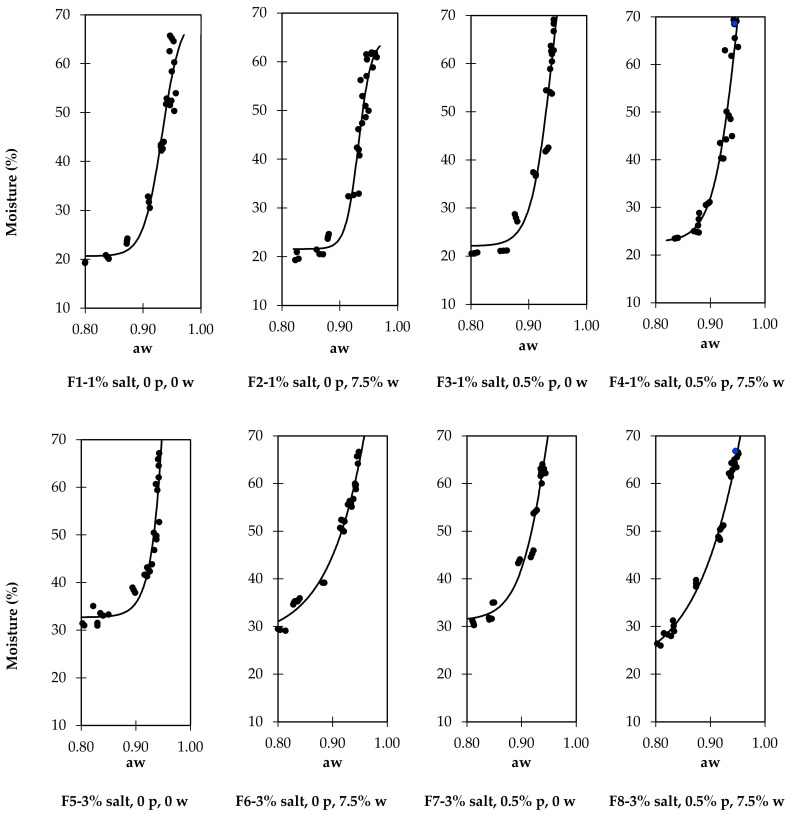
Relation between the water activity (aw) and moisture in cured sausages with different formulations. p—phosphate; w—wine.

The effects of formulation on aw and moisture are presented in Table 2. Figure 2 illustrates the relationship between the moisture and aw for each formulation (upper part, 1% salt; lower part, 3% salt). It is possible to observe a different pattern between sausages with 1% or 3% salt, with the sausages with lower salt having a long segment until nearly 30% moisture, where the moisture reduction has a weak impact on aw. When 3% salt was used, the curve had a different slope, and we observed that the reduction in aw was more concomitant with the reduction in moisture. The relationship between aw and moisture is established by water sorption isotherms [37]. In meat products, the sorption isotherm is type II, having a sigmoid format [38]. Considering the eventual methodological differences between the present results and the sorption isotherms theorized, we can infer that the graphs presented in Figure 2 correspond to the part of the isotherm related to weakly bonded water [39]. That part of the curve has a characteristic high slope once the water involved in this phase becomes free; it occupies the capillary system of the food and is mechanically extractable [40]. Having weak or no bonds to food components, the reduction of aw in this segment depends on water extraction from the food, as happens with drying or adding solutes that bind the water and reduce its availability [41]. The different formats of the curves of dry-cured sausages made with 3% salt might be due to the concentration of salt in the sausage water becoming sufficiently concentrated to have a noticeable impact on aw [25]. The influence of phosphates and wine is shown by the different shapes of curves F7 and F8 and F5 and F6. When both are used (F8), the inflection point of the sigmoid curve occurs only below the lowest aw measured, whereas when no phosphate or wine (F%), or 0.5% phosphate and no wine (F7) is used, the inflection point is detectable at aw values between 0.85 and 0.90. These differences in the isotherm segments presented in Figure 2 follow the difficulty of establishing a relationship between phosphates or wine and the aw discussed above.

These results highlight the importance of exercising caution when modifying the formulation of dry-cured sausages. From the comparison of the four graphs obtained with sausages formulated with 1% added salt with those with higher salt, it is evident that the level of salt has substantial consequences on the drying extension necessary to achieve an aw low enough to consider the product safe [24,42].

**Figure 3:**
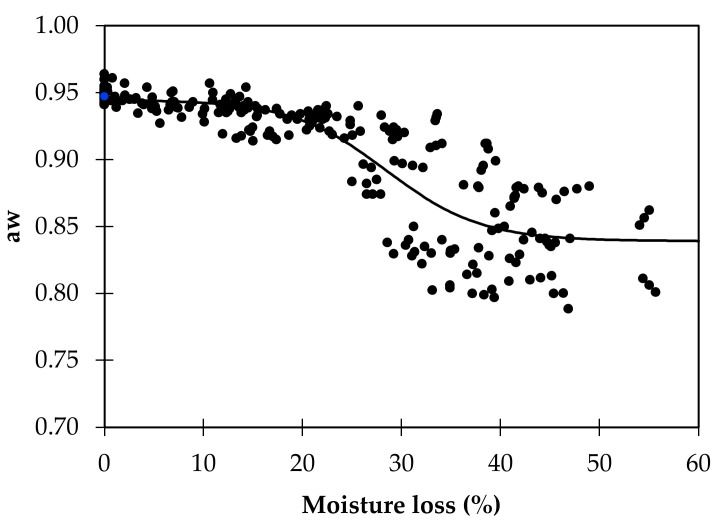
Nonlinear regression between aw and moisture loss for all the formulations (*n* = 240).

**Figure 4:**
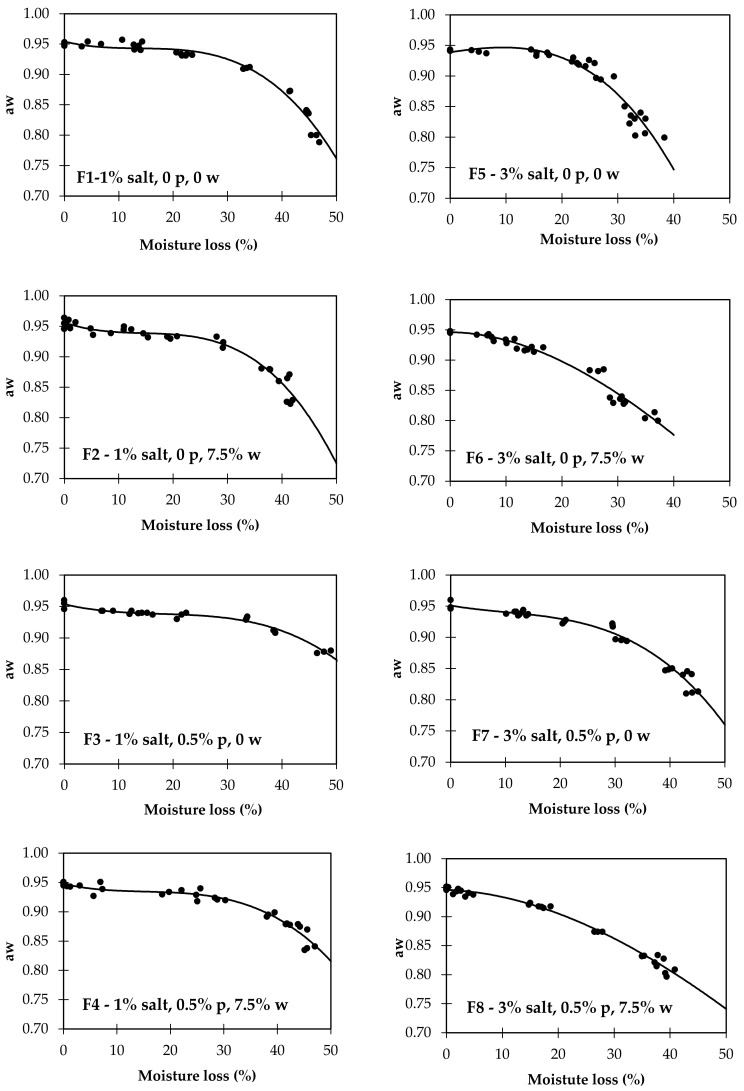
Nonlinear regression between aw and moisture loss for the eight formulations tested (*n* = 30). p—phosphate; w—wine.

The interest of these findings is primarily theoretical, validating the importance of salt in aw reduction, providing new clues on the complex influence that phosphates have on aw, and bringing new insights into the effect of wine on dry-cured sausage aw. Several nonlinear regression functions were tested to establish the relationship between moisture loss and aw, and the best fitting was obtained with the Boltzmann sigmoid function. The determination coefficients evaluated the fitting quality (R^2^), the lowest root mean square error (RMSE), and the visual analysis of the curve. As observed in previous studies, the formulation highly influences the relationship between moisture and aw in dry-cured sausage. Figure 3 and Table 3, where all the data from the eight formulations were used, illustrate the difficulty of establishing the relationship once the interpolation is poor (R^2^ = 0.737), particularly after 20% moisture loss, the phase where the ingredients and additives have a higher impact. The poor quality of this nonlinear regression does not allow us to use it for any estimation purposes. Once the salt level was one of the main effects of the present study, we performed the regression for both levels of salt added separately. The quality of these regressions was also poor. Thus, eight nonlinear regression equations of the curves were calculated (Table 3, Figure 4). The best-fitting was observed for F8 (R^2^ = 0.988, RMSE = 0.006), and the worst-fitting was observed for F3 (R^2^ = 0.933, RMSE = 0.013). These parameters are compatible with estimation purposes [43].

Viewing the evaluation of the predictive ability of each of the eight nonlinear regression equations, we tested the moisture loss data from each formulation to predict aw. The residuals were individually calculated from the 64 combinations (8 equations × 8 sets of moisture loss data) by subtracting the predicted aw from the experimental aw. The percentage of deviation is presented in Table 4 under the heading “present work”. The percentage of deviation of predicted aw using an equation from one formulation and the data used on its nonlinear regression is in the diagonal of the table, highlighted in bold. This percentage of deviation is related to the RMSE for each formulation presented in Table 3 and is, as expected, the lowest of all cases. To predict aw from moisture loss, it is recommended to use models that do not exceed 10% of the deviation [43]. 

The excellent predictive ability for the same formulation is illustrated by the low deviations, lower than 1%. The worst-case possible maximum value detected was consistently below 4%, indicating that the equations do not have a particular problem with extreme cases. When the aw prediction is made with a different formulation equation, the mean values of the deviation are consistently below 10%. The worst case was when data from F3 were used for the aw prediction with Equation F2 (mean percentage of deviation = 8.8%). From the 56 cases of cross prediction (64 minus 8 same-formulation predictions), 25 have a maximum deviation higher than 10%. This high occurrence of incorrect prediction highlights the importance of using data from dry-cured sausages made with a formulation as similar as possible to those used in the prediction model. The importance of using a similar formulation may also be inferred from external data prediction.

We used 169 external sample data on moisture loss and aw of dry-cured sausages from previous works [29,30], which had a base formulation similar to that used in the present work. When the data from one experiment consisted of a survey from small producers [29], the amount of salt was very variable. Thus, we made two groups, one of which was as similar as possible to the formulations of the present work with 1% added salt, considering the sausages that had a salt/moisture ratio of 2% or less. The deviations of the predicted aw values from the experiments are presented in Table 4. These dry-cured sausages were made without phosphates, and most of them were made with wine. Theoretically, the adequate equation would be F2 for those sausages with salt/moisture ≤2 and F6 for those with more salt. The mean deviations were found to be below 10% for all the studied cases, but there is a considerable occurrence of incorrectly predicted values, as inferred by the high maximum deviations observed. In this case, the variations inherent in very different products could have been based on these discrepancies. In addition to the highly variable amounts of salt that were forced into only two groups, the fat level also interfered with the relationship between the moisture and aw, and it was highly variable among the sausages used as external data.

From an applied point of view, the practice equation can be used by the producer of dry-cured sausages to estimate the aw from the moisture loss (Table 5). The producer who uses a formulation such as F4, typical in several small industries, should dry the product until a 35% moisture loss to achieve an aw of 0.91. If a small producer who uses a formulation without phosphates and seasons the sausages with wine (F6) wants to reduce the added salt from 3% for 1% (F2), he must be aware that it is necessary to dry the sausages, not to the usual 20% moisture loss, but to 30%, which is the reduction necessary to achieve an aw of 0.91.

### 3.3. Salmonella Behaviour

**Figure 5:**
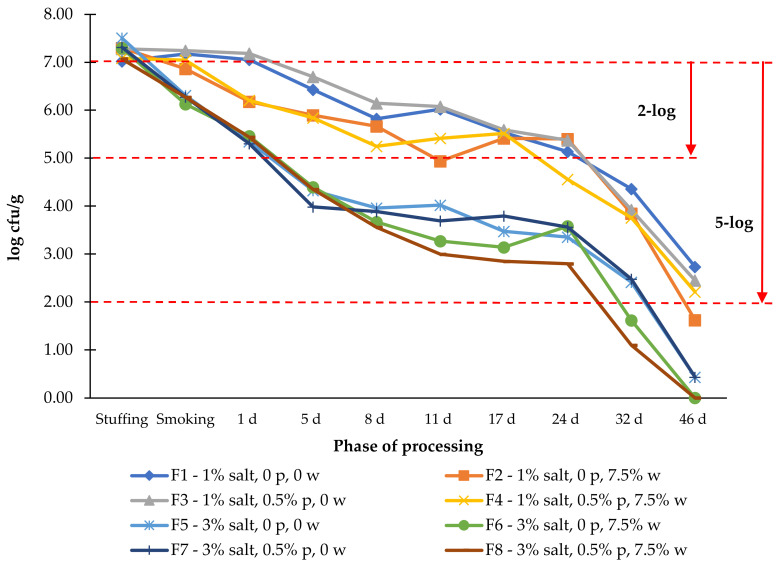
Survival of *Salmonella* during the processing of a cured sausage with the eight formulations tested (*n* = 3). p—phosphate; w—wine.

The *Salmonella* counts in the experimental dry-cured sausages with the different formulations studied in this work are presented in Figure 5.

The initial inoculation was approximately 7 log cfu/g to demonstrate the 5-log reduction proposed by USDA/FSIS [31]. Analyzing the evolution of the pathogen, it is clear that after the smoking phase, the effect of the higher salt level on a faster reduction of the pathogen has a plateau between 8 and 24 days of drying. In 3% salt dry-cured sausages, when wine was used (F6 and F8), a 5-log reduction was achieved at 32 days, while those without wine did not meet that criterion until later in the drying process. At the maximum drying period tested, 46 d, when low salt was used, only the sausages without phosphate and wine achieved a 5-log reduction. These results might seem worrying, as they suggest that extensive drying is necessary to make the product safe, leading to yield and texture adequacy losses. However, an initial count of 7 log cfu/g is theoretical and has no parallel in the reality of a reasonably hygienic industry or workshop. A more realistic amount of initial *Salmonella* contamination might be approximately 2 log cfu/g. With contamination of that order, a 2-log reduction will be sufficient to make the product safe. The 2-log reduction in the data presented here should be carefully interpreted, as it will eventually be biased due to the high initial contamination and consequent competition deregulation among the sausage microbiota. We can observe that when a higher level of salt is used, a 2-log reduction is achieved at five days of drying when the aw is still high (0.93–0.94). When only 1% salt is used, only after 32 d of drying is the 2-log reduction met, with the sausages already having a very low aw (0.83–0.87). The lethality observed in the present work was lower than that observed with a similar product by several authors [44,45,46].

From the analysis of Figure 5, the effect of the drying and salt level is clear, and the effect of phosphates and wine does not seem to have a pattern. To evaluate the contribution of each parameter to the desired 5- or 2-log reduction in the *Salmonella* population, we made a logistic regression model to predict the contribution of aw, salt, phosphates, and wine to meet the reduction criteria (Table 6). The reference class was the lower amount or absence of categorical variables (salt, phosphates, and wine). The continuous variable aw had to be modified (multiplied by 100) once the logistic regression algorithm assumed an increase of one unit in the predictor, and the aw range was only one unit.

**Figure 6:**
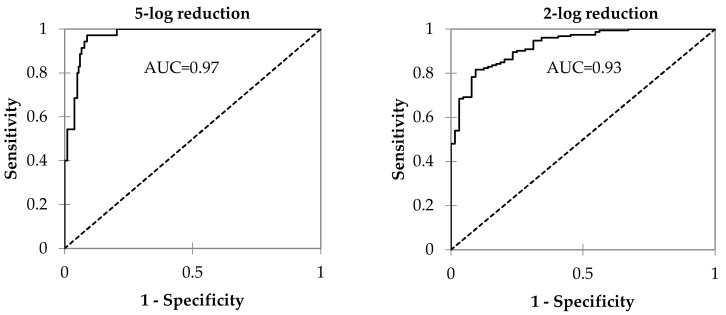
Receiver operating characteristic curves (ROC) of logistic regression models to predict the *Salmonella* population’s 5- and 2-log reduction.

Both models have good predictive ability, as revealed by the high area under the curve (AUC) of the receiver operating characteristic curves (ROC) (Figure 6). For the 5-log reduction, the AUC was 0.97, and for the 2-log reduction, it was slightly lower (0.93). As the AUC approaches 1, the model’s prediction ability improves, indicating that most of the events will be correctly predicted [47].

The logistic model for a 5-log reduction shows that for one unit of increment in aw − 100 (one centesimal in the real value), the odds of reaching the previewed reduction are reduced to half (OR = 0.51). Using 3% salt instead of 1% corresponded to a 7.49-fold increase in achieving the criteria. The use of phosphate was not significant, indicating that it has no impact in this model. The presence of wine is not significant (*p* = 0.062), but exploring the trend, once the effect of wine is not well-known, we can see that it has OR = 3.53, suggesting a contribution for the 5-log reduction. The pattern for the 2-log reduction is very similar. In this case, once this reduction was calculated between 7 and 5 log cfu/g, where the products still had high moisture, the effect of the salt was more pronounced, with an OR = 14.72. The wine in this phase was significant, indicating that sausages prepared with wine have a 2.56-fold higher probability of reaching the criteria.

## 4. Conclusions

The formulation of the dry-cured sausage influences its moisture content and aw. While the effect of the salt level was very evident, the presence of wine and phosphates had only minimal effects, and it was not possible to extract a trend of its influence on the aw. In addition, when used together, it might be expected that they nullify each other’s effect. Viewing the evaluation of the predictive ability of each of the eight nonlinear regression equations, we tested the moisture loss data from each formulation to predict aw. The predictive ability was excellent for the same formulation, with deviations between 1% and 4%. When the aw prediction was made with a different formulation equation, the mean deviation was below 10%, an acceptable deviation, in 82% of the situations. Data from external samples (not used in the estimation) were also used, and the results were compatible with the prediction.

From an applied point of view, the presented estimation from the moisture loss can be used by a producer to estimate the aw for a specific formulation, particularly the salt level. Reducing salt addition from 3% to 1% has substantial repercussions on *Salmonella* survival through its effect on aw. With 1% salt, the safety of *chouriço* is achieved but with a more extended drying period, which has consequences on the yield.

Even though aw is not considered a hurdle with an expected lethal effect, as is heating, we observed that it is possible to use that hurdle to eliminate this pathogen at levels of sensorially accepted aw.

The current trend to reduce salt in foods must be carefully approached if applied to cured meat products, as it has substantial consequences on aw evolution and *Salmonella* survival.

## Figures and Tables

**Table 1:** Experimental design.

Variable Ingredients	F1	F2	F3	F4	F5	F6	F7	F8
Salt	1%	1%	1%	1%	3%	3%	3%	3%
Phosphate (E451 + E4521)	-	-	0.5%	0.5%	-	-	0.5%	0.5%
Wine	- ^1^	7.5%	- ^1^	7.5%	- ^1^	7.5%	- ^1^	7.5%

^1^ The same amount of 7.5% water was used.

**Table 2:** Water activity (aw) and moisture in cured sausage with different formulations during processing. Results expressed as mean ± standard deviation. (*p* = phosphate).

Phase of Processing	1% Salt				3% Salt				*p* Value (Main Effects)		
	No Phosphate		0.5% Phosphate		No Phosphate		0.5% Phosphate		Salt	*p*	Wine
	No Wine	7.5% Wine	No Wine	7.5% Wine	No Wine	7.5% Wine	No Wine	7.5% Wine			
aw											
Stuffing	0.95 ± 0.00 a	0.96 ± 0.01 a	0.95 ± 0.01 a	0.95 ± 0.00 a	0.94 ± 0.00 a	0.95 ± 0.00 a	0.95 ± 0.01 a	0.95 ± 0.00 a	0.053	0.335	0.863
Smoking	0.95 ± 0.00 ab	0.95 ± 0.01 a	0.94 ± 0.00 bc	0.94 ± 0.00 bc	0.94 ± 0.00 c	0.94 ± 0.00 bc	0.94 ± 0.00 bc	0.95 ± 0.00 abc	0.002	0.104	0.018
1 d drying	0.95 ± 0.01 a	0.95 ± 0.01 a	0.94 ± 0.00 a	0.94 ± 0.01 a	0.94 ± 0.00 a	0.94 ± 0.00 a	0.94 ± 0.00 a	0.94 ± 0.00 a	0.148	0.295	0.886
5 d drying	0.95 ± 0.01 a	0.94 ± 0.00 ab	0.94 ± 0.00 abc	0.93 ± 0.00 bc	0.93 ± 0.00 bc	0.93 ± 0.00 c	0.94 ± 0.00 abc	0.94 ± 0.00 abc	0.002	0.136	0.222
8 d drying	0.93 ± 0.00 abc	0.94 ± 0.01 a	0.94 ± 0.00 ab	0.93 ± 0.01 abc	0.92 ± 0.00 abc	0.92 ± 0.00 c	0.93 ± 0.00 abc	0.92 ± 0.00 bc	<0.001	0.667	0.262
11 d drying	0.93 ± 0.00 a	0.93 ± 0.00 a	0.93 ± 0.00 a	0.92 ± 0.00 b	0.92 ± 0.00 b	0.92 ± 0.00 b	0.92 ± 0.00 b	0.92 ± 0.00 b	<0.001	0.009	0.003
17 d drying	0.91 ± 0.00 b	0.92 ± 0.01 a	0.91 ± 0.00 b	0.90 ± 0.00 c	0.90 ± 0.00 c	0.88 ± 0.00 d	0.90 ± 0.00 c	0.87 ± 0.00 d	<0.001	<0.001	<0.001
24 d drying	0.87 ± 0.00 a	0.88 ± 0.00 a	0.88 ± 0.00 a	0.88 ± 0.00 a	0.84 ± 0.01 bc	0.84 ± 0.00 cd	0.85 ± 0.00 b	0.83 ± 0.00 d	<0.001	0.214	0.045
32 d drying	0.84 ± 0.00 cd	0.87 ± 0.00 ab	0.86 ± 0.00 b	0.87 ± 0.00 a	0.83 ± 0.00 de	0.83 ± 0.00 cde	0.84 ± 0.00 c	0.82 ± 0.01 e	<0.001	<0.001	0.002
46 d drying	0.80 ± 0.01 c	0.83 ± 0.00 a	0.81 ± 0.00 bc	0.84 ± 0.00 a	0.80 ± 0.00 bc	0.81 ± 0.01 bc	0.81 ± 0.00 b	0.80 ± 0.00 bc	<0.001	0.003	<0.001
Moisture											
Stuffing	65.18 ± 0.46 c	61.46 ± 0.40 d	75.69 ± 0.43 a	69.44 ± 0.86 b	65.84 ± 1.08 c	65.49 ± 1.02 c	74.77 ± 0.52 a	66.18 ± 0.54 c	0.721	<0.001	<0.001
Smoking	60.43 ± 1.68 c	61.27 ± 0.57 bc	68.07 ± 1.00 a	67.80 ± 1.63 a	60.70 ± 1.10 c	59.38 ± 0.49 c	63.07 ± 0.76 bc	64.76 ± 1.31 ab	0.001	<0.001	0.690
1 d drying	53.09 ± 0.66 c	57.39 ± 1.09 b	63.05 ± 0.48 a	62.83 ± 0.77 a	50.71 ± 1.49 c	57.41 ± 1.69 b	62.46 ± 0.44 a	64.30 ± 0.57 a	0.468	<0.001	<0.001
5 d drying	51.19 ± 0.62 c	51.30 ± 1.26 c	60.46 ± 1.25 a	49.33 ± 0.63 cd	46.79 ± 2.43 d	55.68 ± 0.51 b	61.29 ± 0.96 a	62.12 ± 0.59 a	<0.001	<0.001	0.593
8 d drying	43.73 ± 0.22 d	47.39 ± 1.01 c	54.13 ± 0.28 a	44.26 ± 0.59 d	42.72 ± 0.35 d	52.20 ± 0.13 b	54.05 ± 0.29 a	50.77 ± 0.37 b	<0.001	<0.001	1.000
11 d drying	42.62 ± 0.30 d	41.74 ± 0.67 d	42.17 ± 0.34 d	40.31 ± 0.03 e	41.47 ± 0.14 de	50.32 ± 0.29 a	45.22 ± 0.56 c	48.50 ± 0.26 b	<0.001	0.934	<0.001
17 d drying	31.68 ± 0.94 de	32.68 ± 0.21 d	37.06 ± 0.30 c	30.82 ± 0.24 e	38.38 ± 0.43 bc	39.16 ± 0.02 b	43.64 ± 0.32 a	39.03 ± 0.59 b	<0.001	<0.001	<0.001
24 d drying	23.73 ± 0.42 e	24.18 ± 0.39 e	27.97 ± 0.61 d	27.52 ± 1.07 d	33.29 ± 0.20 b	35.58 ± 0.27 a	34.99 ± 0.01 ab	30.12 ± 0.90 c	<0.001	0.005	0.043
32 d drying	20.47 ± 0.30 e	20.85 ± 0.45 e	21.15 ± 0.04 e	24.87 ± 0.10 d	32.49 ± 1.83 b	34.95 ± 0.29 a	31.60 ± 0.14 b	28.28 ± 0.25 c	<0.001	0.055	0.032
46 d drying	18.99 ± 0.48 d	19.97 ± 0.71 d	20.65 ± 0.10 d	23.55 ± 0.05 c	30.39 ± 1.13 a	29.27 ± 0.16 a	30.70 ± 0.38 a	26.38 ± 0.35 b	<0.001	0.025	0.171

a, b, c, d, e. To compare formulations, in the same line, means followed by different letters are significantly different (*p* < 0.05).

**Table 3:** Parameters of the nonlinear regression for each formulation and for the total data.

Formulation (*n* = 30)	Boltzmann Sigmoid Aw = a + (b − a)/(1 + exp((c − ML)/d))				R^2^	RMSE
	a	b	c	d		
F1-1% salt, 0 p, 0 w	0.948	−73.103	100.966	8.747	0.980	0.008
F2-1% salt, 0 p, 7.5% w	0.952	−28.335	100.553	10.577	0.941	0.011
F3-1% salt, 0.5% p, 0 w	0.948	−15.601	119.355	13.058	0.933	0.013
F4-1% salt, 0.5% p, 7.5% w	−19.618	0.945	108.252	−11.584	0.942	0.009
F5-3% salt, 0 p, 0 w	0.939	0.792	29.897	2.899	0.960	0.011
F6-3% salt, 0 p, 7.5% w	0.951	0.734	30.092	8.312	0.969	0.009
F7-3% salt, 0.5% p, 0 w	0.949	0.638	47.813	9.845	0.965	0.009
F8-3% salt, 0.5% p, 7.5% w	0.734	0.955	33.096	−10.302	0.988	0.006
Total (*n* = 240)	0.945	0.839	28.470		0.737	0.025

ML—moisture loss; R—regression coefficient; RMSE—root mean squares of the errors; p—phosphates; w—wine.

**Table 4:** Percentage of deviation obtained in the prediction of aw using the eight equations obtained from each formulation. Data used as predictors are from the present work and external data. The results are presented as the mean (minimum-maximum) of the percentage of deviation.

Experiment	Predicted from Each Formulation							
	F1	F2	F3	F4	F5	F6	F7	F8
Present work								
F1-1% salt, 0 p, 0 w ^1^	0.7 (0.0–2.5)	1.3 (0.0–4.2)	2.7 (0.0–12.1)	1.4 (0.0–6.9)	3.0 (0.0–10.1)	4.9 (0.2–10.9)	1.3 (0.1–3.6)	3.6 (0.1–8.2)
F2-1% salt, 0 p, 7.5% w	1.3 (0.0–5.4)	0.9 (0.0–3.2)	2.4 (0.0–10.0)	1.6 (0.1–7.0)	3.1 (0.0–8.9)	4.2 (0.1–10.6)	1.0 (0.0–3.3)	3.0 (0.0–7.9)
F3-1% salt, 0.5% p, 0 w	8.1 (0.1–34.7)	8.8 (0.0–34.8)	0.9 (0.0–3.9)	3.2 (0.1–14.2)	4.6 (0.0–12.4)	6.9 (0.1–14.3)	4.3 (0.0–14.3)	5.7 (0.0–12.2)
F4-1% salt, 0.5% p, 7.5% w	1.5 (0.0–6.2)	2.6 (0.0–9.2)	1.7 (0.1–6.8)	0.7 (0.0–2.7)	4.5 (0.0–11.3)	6.4 (0.0–12.5)	2.3 (0.0–6.8)	5.0 (0.1–10.0)
F5-3% salt, 0 p, 0 w	4.3 (0.1–14.2)	3.5 (0.0–12.4)	4.6 (0.1–15.3)	3.9 (0.1–13.9)	0.8 (0.0–3.2)	2.3 (0.0–5.7)	2.8 (0.1–11.1)	2.0 (0.1–5.2)
F6-3% salt, 0 p, 7.5% w	4.8 (0.1–13.1)	4.3 (0.2–11.1)	5.1 (0.0–14.7)	4.5 (0.1–13.0)	1.7 (0.0–5.4)	0.7 (0.0–2.9)	3.6 (0.0–9.8)	1.2 (0.0–4.3)
F7-3% salt, 0.5% p, 0 w	1.6 (0.1–5.0)	1.1 (0.0–3.3)	3.3 (0.0–11.2)	2.1 (0.0–7.7)	2.7 (0.0–6.3)	4.3 (0.1–8.8)	0.7 (0.0–2.6)	3.0 (0.0–6.4)
F8-3% salt, 0.5% p, 7.5% w	4.1 (0.1–10.8)	3.3 (0.1–8.2)	4.9 (0.0–14.4)	4.1 (0.1–11.9)	1.6 (0.0–3.9)	1.3 (0.0–4.6)	2.7 (0.0–7.4)	0.5 (0.0–1.8)
External data								
Lower salt level (salt/moisture ≤ 2) ^1^	2.7 (0.0–20.4)	3.6 (0.1–22.7)	2.9 (0.0–19.3)	2.3 (0.0–13.9)	8.6 (0.3–13.2)	9.6 (0.5–15.7)	3.8 (0.1–13.5)	7.5 (0.5–13.8)
Higher salt level (salt/moisture > 2) ^2^	7.5 (0.0–25.5)	7.4 (0.1–26.3)	10.0 (0.0–28.2)	6.9 (0.2–23.6)	5.1 (0.0–15.7)	6.0 (0.0–15.5)	5.3 (0.2–18.1)	5.0 (0.0–13.3)

^1^*n* = 30; ^2^ *n* = 79.

**Table 5:** Prediction of aw from possible moisture losses followed by the industry, using the eight equations obtained from each formulation.

Moisture Loss	Aw Predicted from Each Formulation							
	F1	F2	F3	F4	F5	F6	F7	F8
20%	0.94	0.94	0.94	0.94	0.93	0.90	0.93	0.91
25%	0.94	0.93	0.94	0.93	0.92	0.87	0.92	0.89
30%	0.93	0.91	0.93	0.92	0.86	0.84	0.90	0.86
35%	0.91	0.89	0.92	0.91	0.81	0.81	0.88	0.83

**Table 6:** The logistic regression model results for achieving a 5- and 2-log reduction in the *Salmonella* population in a cured sausage, considering the aw, salt level, phosphates, and wine as predictors.

Variable	Beta	SE ^1^	Wald χ^2^	*p*-Value	OR ^2^	95% CI ^3^
5-log reduction						
Intercept	54.33	10.37	27.46	<0.0001		
aw × 100	−0.68	0.13	28.26	<0.0001	0.51	0.39–0.65
Salt, 3%	2.01	0.68	8.77	0.003	7.49	1.98–28.36
Phosphate, 0.5%	0.97	0.66	2.17	0.141	2.63	0.73–9.49
Wine, 7.5%	1.26	0.68	3.49	0.062	3.53	0.94–13.26
2-log reduction						
Intercept	61.28	11.58	28.02	<0.0001		
aw × 100	−0.67	0.12	28.74	<0.0001	0.51	0.40–0.65
Salt, 3%	2.69	0.48	31.86	<0.0001	14.72	5.79–37.44
Phosphate, 0.5%	−0.62	0.45	1.93	0.165	0.54	0.22–1.29
Wine, 7.5%	0.94	0.45	4.32	0.038	2.56	1.06–6.19

^1^ SE: standard error; ^2^ OR: Odds ratio; ^3^ CI: Confidence Interval.
